# p27^Kip1^ and p21^Cip1^ collaborate in the regulation of transcription by recruiting cyclin–Cdk complexes on the promoters of target genes

**DOI:** 10.1093/nar/gkv593

**Published:** 2015-06-13

**Authors:** Serena Orlando, Edurne Gallastegui, Arnaud Besson, Gabriel Abril, Rosa Aligué, Maria Jesus Pujol, Oriol Bachs

**Affiliations:** 1 Department of Cell Biology, Immunology and Neurosciences, University of Barcelona, 08036-Barcelona, Spain; 2 INSERM UMR1037, Cancer Research Center of Toulouse, Toulouse, France; 3 Université de Toulouse, Toulouse, France; 4 CNRS ERL5294, Toulouse, France

## Abstract

Transcriptional repressor complexes containing p130 and E2F4 regulate the expression of genes involved in DNA replication. During the G_1_ phase of the cell cycle, sequential phosphorylation of p130 by cyclin-dependent kinases (Cdks) disrupts these complexes allowing gene expression. The Cdk inhibitor and tumor suppressor p27^Kip1^ associates with p130 and E2F4 by its carboxyl domain on the promoters of target genes but its role in the regulation of transcription remains unclear. We report here that p27^Kip1^ recruits cyclin D2/D3–Cdk4 complexes on the promoters by its amino terminal domain in early and mid G_1_. In cells lacking p27^Kip1^, cyclin D2/D3–Cdk4 did not associate to the promoters and phosphorylation of p130 and transcription of target genes was increased. In late G_1_, these complexes were substituted by p21^Cip1^-cyclin D1–Cdk2. In p21^Cip1^ null cells cyclin D1–Cdk2 were not found on the promoters and transcription was elevated. In p21/p27 double null cells transcription was higher than in control cells and single knock out cells. Thus, our results clarify the role of p27^Kip1^ and p21^Cip1^ in transcriptional regulation of genes repressed by p130/E2F4 complexes in which p27^Kip1^ and p21^Cip1^ play a sequential role by recruiting and regulating the activity of specific cyclin–Cdk complexes on the promoters.

## INTRODUCTION

Cyclin-dependent kinases (Cdks) are serine/threonine kinases characterized by their need for associating with a regulatory subunit, the cyclin that modifies Cdk conformation and provides domains necessary for catalytic activity (1). This family of kinases includes 20 members named Cdk1 through Cdk20 (2). Many members of this family, including Cdk4, Cdk6, Cdk2 and Cdk1, are involved in cell-cycle regulation (3). Cdk4, Cdk6 and Cdk2 regulate progression through the G_1_ phase although additionally, Cdk2 also regulates S phase. Finally, Cdk1 regulates mitosis. Binding of Cdks to specific cyclins confers a functional specialization to each complex (3). In response to mitogenic stimuli, the synthesis of the D-type cyclins is induced in early–mid G_1_ phase. These cyclins associate with Cdk4 and Cdk6, forming complexes that phosphorylate and inactivate members of the retinoblastoma family of pocket proteins (pRb, p107 and p130) (4). These adaptor proteins form complexes with E2Fs that repress transcription. Phosphorylation of pocked proteins by cyclin D-Cdk4/6 primes them for further phosphorylation by Cdk2 at other sites (5). These phosphorylations inactivate the pocket proteins resulting in de-repression of multiple genes encoding for proteins required for DNA replication (S phase) or mitosis. In addition to the regulation by cyclins, Cdk activity is also regulated by other mechanisms that include phosphorylation of specific amino acid residues, acetylation and binding to proteins called Cdk-inhibitors (CKIs) (1,2,6,7).

Two families of CKIs have been described. One is the Cip/Kip family that includes p21^Cip1^ (p21), p27^Kip1^ (p27) and p57^Kip2^ (p57) that associate with most cyclin–cdk complexes (8). The second is the INK4 family that includes p15^INK4B^, p16^INK4A^, p18^INK4C^ and p19^INK4D^ that specifically acts on Cdk4 and Cdk6 (9). All members of the Cip/Kip family of CKIs interact with both cyclin and Cdk subunits by two specific sequences in the Kinase Inhibitory Domain (KID), included in the NH_2_-region. The mechanism of inhibition consists in introducing a portion of the Cdk-binding domain into the catalytic center of the kinase, thus preventing interaction of the Cdk with ATP (10). Interestingly, it has been shown that the specific phosphorylation of two tyrosine residues of p27 (Y74 and Y88) in the NH_2_ domain, by members of the Src tyrosine kinase family, induces a conformational change that modifies the interaction of p27 with the catalytic center of the Cdk (11–13) inducing a partial activation of the kinase (14). Thus, under these circumstances cyclin–Cdk complexes might be partially active despite its association with p27. Similarly, p21 can be phosphorylated at Y76 (that is equivalent to Y88 in p27) by Src family members. This phosphorylation also reduces the inhibitory capability of p21 and as a consequence the cyclin–Cdk complexes associated with Y76-phosphorylated p21 are partially active (15).

The carboxyl moieties of p27 and p21 are considered intrinsically disordered domains that can adopt different conformations depending on the protein partners that associate with these regions (16). This characteristic probably underlies the large spectra of cellular functions that can be performed by Cip/Kip proteins. Among these functions there is the regulation of transcription. Data from ChIP-on-chip revealed that p27 associates with specific gene promoters (p27-target genes, p27-TGs) (17). The analysis of these promoters indicated that they are enriched with sequences that interact with E2F4. Subsequent analysis revealed that p27 directly interacts with p130 and E2F4 by its carboxyl moiety and that it acts as a transcriptional co-repressor (17). p21 is also involved in transcriptional regulation. It has been reported to behave as a transcriptional repressor by associating with a number of transcription factors such as E2Fs, Myc, NRf2, CBP and STAT3 among others (18–20).

Interestingly, two recent papers demonstrated that p27 (21) and p21 (22) regulate the expression of Sox2. These reports describe that p27 and p21 associate with the same Sox2-SRR2 enhancer and that their association represses the expression of Sox2. In the case of p27 repression was mediated by association with p130/E2F4 complexes. Even though in the case of p21 the participation of p130/E2F4 complexes has not been studied, these results suggest that p27 and p21 can regulate Sox2 expression by similar mechanisms on the same regulatory region of the chromatin.

We report here that p27 and p21 collaborate in the transcriptional regulation of p130/E2F4-dependent genes during cell cycle progression. Specifically, we demonstrate that on the promoters of specific target genes (Aurka and Med18), p27 recruits cyclin D2/D3–Cdk4 complexes necessary for the phosphorylation of p130 in mid G_1_. Subsequently, these complexes are substituted by p21-cyclin D1–Cdk2 complexes needed for further phosphorylation of p130 in late G_1_, hence inducing the full transcription of target genes. Thus, p27 and p21 located on the promoters play a key role in controlling the timing of gene transcription in G_1_ and in the subsequent progression through the cell division cycle by recruiting specific cyclin–Cdk complexes to these promoters.

## MATERIALS AND METHODS

### Cell culture and lentiviral infection

Mouse embryonic fibroblasts (MEFs) were cultured in DMEM (Dulbecco's modified Eagle Medium) supplemented with 10% fetal bovine serum whereas NIH3T3 cells were cultured in DMEM with 10% donor bovine serum. In addition, all cell lines were supplemented with 2 mM L-Glutamine, 1% non-essential amino acids, 1 mM pyruvic acid, 50 U/ml penicillin and 50 μg/ml streptomycin. Cultures were maintained at 37ºC and 5% CO_2_. p27 KO or p21 KO MEFs were infected with the lentiviral plasmid pLV-Ires-GFP containing full length p27 or p21. The protocol for viral particles production and cell infections has been described elsewhere (23). Forty-eight hours after infection, cells were serum deprived for 3 days. Then, they were collected and subjected to western blot (WB), RNA isolation and qPCR analysis.

### Cell synchronization

MEFs were maintained in serum free medium for 72 h whereas NIH3T3 cells were serum starved for 48 h. Activation of proliferation was induced by serum addition. Cells were collected at several times: at 6 h (NIH3T3) or 8 h (MEFs) that correspond to mid- G_1_ phase and at 12 h (NIH3T3) or 18 h (MEFs) corresponding to G_1_/S phase transition. Cell synchronization was monitored by flow cytometry of propidium iodide-stained cells. The cell cycle timings upon serum stimulation in p27^−/−^, p21^−/−^ and DKO (p21 and p27 null cells)–MEFs were similar to that of wild-type (WT) MEFs (24). Thus, cells from these different cultures were collected at the same times that WT-MEFs

### Flow cytometry analysis

Cells were fixed with 70% cold ethanol for 2 h at 4ºC, washed with phosphate buffered saline (PBS) and incubated with 20 μg/ml of propidium iodide and 200 μg/ml RNase for 30 min at room temperature. Analysis of DNA content was carried out in a Becton Dickinson FACS Calibur. Data were analyzed with the WinMDI 2.9 software.

### Immunoprecipitation

NIH3T3 cells and MEFs were scraped and washed twice with PBS. Pellets were lysed in 2 ml of immunoprecipitation (IP) buffer (PBS containing 0.5% Triton X-100, 1 mM EDTA, 100 μM sodium orthovanadate, 0.25 mM phenylmethylsulfonyl fluoride, complete protease inhibitor mixture by Roche Applied Science and 1/25 vol of DNAse I). Cell lysates were incubated on ice for 30 min and then centrifuged at 3000 rpm at 4°C for 5 min. Protein concentration was determined by Bradford protein assay using Bradford reagent (BioRad). Thirty microgram of protein extract was kept as input sample and 1 mg of total protein was incubated overnight with 2 μg of antibody. Antibodies used for IP were the same than those used for chromatin IP (ChIP) experiments. Magnetic beads (Dynabeads, Invitrogen) were added and samples were incubated in rotation for 2 h at 4ºC. Beads were washed five times with lysis buffer, eluted with 0.1 M Citrate pH 2.5 and boiled in Laemmli buffer for WB analysis. As a control, lysates were incubated with irrelevant IgG.

### ChIP

Cells were grown to confluence and synchronized in quiescence or at different times of G_1_ phase. ChIP assays were performed as previously described (25). Briefly, cells were lysed and chromatin from cross-linked cells was sonicated. Chromatin was incubated with 2.5–5 μg of antibodies against p27 (sc-528, Santa Cruz), p21 (sc-397), cyclin D1 (sc-246), cyclin D2 (sc-181), cyclin D3 (sc-182), Cdk4 (sc-260) or Cdk2 (sc-6248) in RIPA buffer, adding 20 μl of Magna ChIP Protein A magnetic beads (Millipore). Samples were incubated in rotation overnight at 4ºC. Beads were washed with low salt buffer, high salt buffer, LiCl buffer and TE buffer. Subsequent elution and purification of the immunoprecipitated DNA–proteins complexes was performed using the IPure kit (Diagenode) according to manufacturer's protocol. Samples were analyzed by quantitative PCR (qPCR). Primer sequences used for qPCR were listed in Supplementary Table S1.

### RNA extraction, reverse transcription-PCR and qPCR for gene expression analysis

Total RNA from MEFs was extracted using High Pure RNA Isolation kit (Roche). cDNA was obtained by reverse transcription-PCR from 1 μg of RNA using SuperScript ViLO cDNA synthesis (Invitrogen) according to manufacturer's instructions. Gene expression was analyzed by real-time qPCR using LightCycler 480 SYBR green I master mix (Roche), corrected by actin or GAPDH expression and expressed as relative units. Primer sequences used for qPCR were listed in Supplementary Table S1.

### WB antibodies

Antibodies against cyclin D1 (sc-246), cyclin D2 (sc-181), cyclin D3 (sc-182), Cdk2 (sc-163), Cdk4 (sc-260) and p21 (sc-6246) were purchased from Santa Cruz. p27 antibody (610242 BD) was purchased from BD Transduction Laboratories. Actin antibody (0869100) was obtained from MP Biomedicals. p130-phospho antibody (2272–1) was purchased from Epitomics and tubulin antibody (T5192) was obtained from Sigma-Aldrich.

## RESULTS

### p27 associates with the promoters of p27-target genes in early and mid G_1_ phase

**Figure 1.**
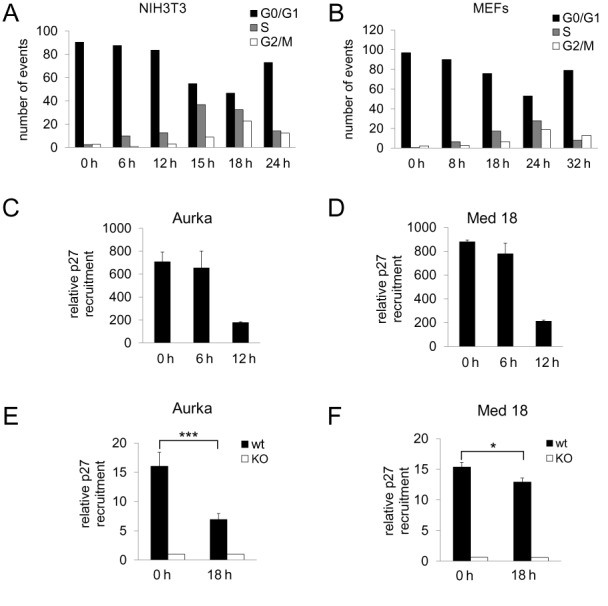
Association of p27 with the promoters of target genes. Flow cytometry analysis of NIH3T3 cells (**A**) or MEFs (**B**) performed at different times after proliferative activation. The association of p27 with the promoter of the Aurka (**C**) or Med18 genes (**D**) in NIH3T3 cells was analyzed by ChIP using anti-p27 or without antibodies as a control. Results are the mean value ± SD of three independent experiments and are relative to the control. The association of p27 with the promoter of the Aurka (**E**) or Med18 genes (**F**) in p27^wt^ and p27^KO^ MEFs was analyzed by ChIP using anti-p27 or without antibodies as a control. Results are the mean value ± SD of three independent experiments and are relative to the control. Statistical analysis was performed using the Student's t-test. *P < 0.05, ***P < 0.001.

We first defined the timing of cell cycle progression in NIH3T3 cells. Cells were serum starved for 48 h and proliferation was induced by serum addition. As shown in Figure 1A, S phase started at 6 h and maximal DNA synthesis was at 15 h. Cell cycle progression analysis in MEFs revealed that DNA replication started at 8 h being the maximal DNA synthesis at 24 h (Figure 1B). Once timing was defined, we studied the association of p27 with the promoters of two p27-TGs, Aurka and Med18 (17), by ChIP at different times after proliferative activation of NIH3T3 cells and MEFs. We selected the times corresponding to G_0_ (0 h) mid G_1_ (6 h for NIH3T3 and 8 h for MEFs) and late G_1_ (12 h for NIH3T3 and 18 h for MEFs). Results revealed that in NIH3T3 cells the association of p27 with the promoters was high in G_0_ and mid G_1_ and significantly decreased in late G_1_ (Figure 1C and D). Similar results were observed in control MEFS. In this case, p27^−/−^-MEFs were used as a negative control (Figure 1E and F).

### Differential association of D-type cyclins with the promoters of p27 target genes

**Figure 2.**
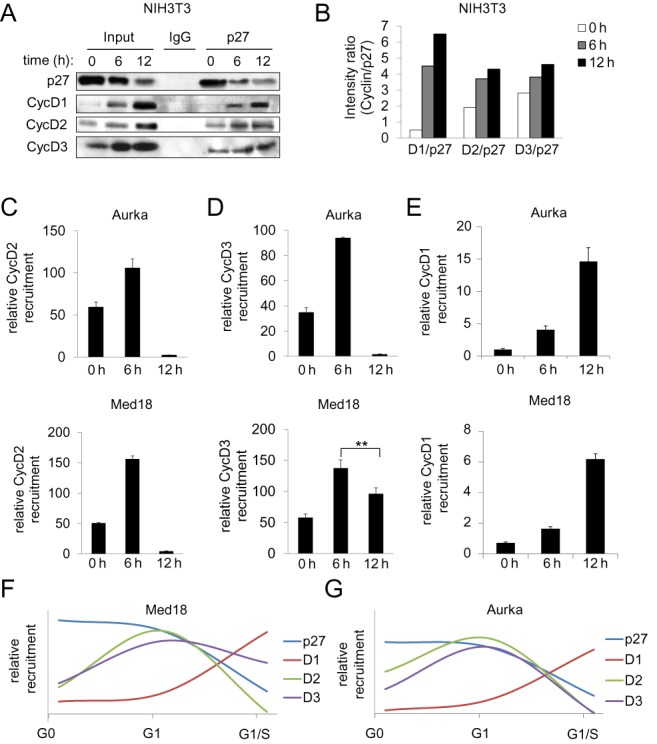
Differential association of D-type cyclins with the promoters of target genes. (**A**) The levels of p27 and D-type cyclins, at different times after proliferative activation of NIH3T3 cells, were determined by WB (input). The association of p27 with the D-type cyclins was analyzed by IP using anti-p27. IP using a non-specific IgG was used as a control. (**B**) Quantification of IP experiments. Results are the mean value of three independent experiments and are relative to p27 levels. The association of cyclin D2 (**C**), cyclin D3 (**D**) and cyclin D1 (**E**) with the Aurka promoter (upper panels) or the Med18 promoter (bottom panels) in NIH3T3 cells was analyzed by ChIP using anti-p27 or without antibodies as a control. Results are the mean value ± SD of three independent experiments and are relative to the control. Graphics showing the association kinetics of p27, cyclin D1, D2 and D3 to the Med18 (**F**) and Aurka (**G**) promoters. Statistical analysis was performed using the Student's t-test. **P < 0.01.

The levels of p27 and D-type cyclins at different times after proliferative activation of NIH3T3 cells were determined by WB. Figure 2A shows that the amount of p27 decreased from quiescence till late G_1_ whereas that of cyclins D1, D2 and D3 increased during the same period of time. To analyze the association of p27 with the different D-type cyclins, IP experiments using anti-p27 were performed. In G_0_, p27 associated with cyclin D2 and D3 but not with cyclin D1. At 6 h p27 associated with all three D-type cyclins and this association increased in late G_1_ (Figure 2A). Quantification of three different experiments was represented in a graph (Figure 2B). The association of D-type cyclins with the p27-TG promoters was then determined by ChIP. As shown in Figure 2C and D, cyclins D2 and D3 behave similarly. Their association with the Aurka and Med18 promoters was high in G_0_ and further increased in mid G_1_ to subsequently decrease in late G_1_. Conversely, the association of cyclin D1 with the promoters was quite different. It was low in quiescent cells, increased in mid G_1_ and was high in late G_1_ (Figure 2E). Figure 2F and G summarize the association kinetics of p27 and cyclins D1, D2 and D3 with the promoters in NIH3T3 cells.

### p27 is necessary for the association of cyclins D2 and D3 with the promoters of target genes

**Figure 3.**
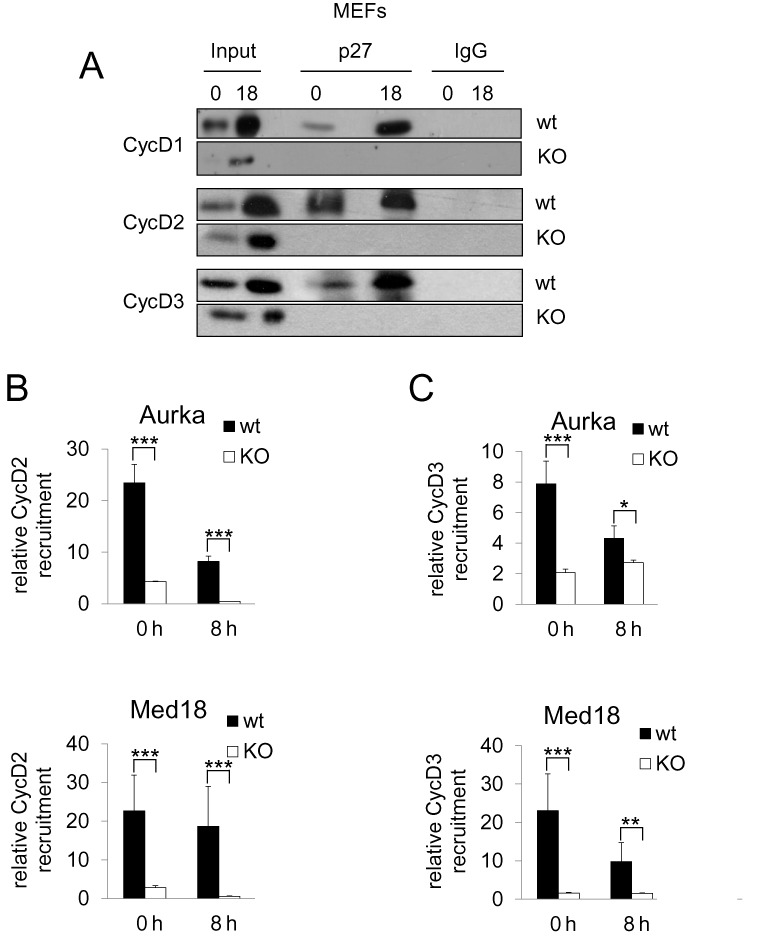
p27 is required for the recruitment of cyclin D2 and D3 on the promoters of target genes in early and mid G_1_. (**A**) The association of p27 with the D-type cyclins at different times after proliferative activation of MEFs was analyzed by IP using anti-p27. IP using a non-specific IgG was used as a control. The association of cyclin D2 (**B**) and cyclin D3 (**C**) with the Aurka promoter (upper panels) or the Med18 promoter (bottom panels) in p27^wt^ and p27^−/−^ MEFs was analyzed by ChIP using anti-cyclin D2, anti-cyclin D3 or without antibodies as a control. Results are the mean value ± SD of three independent experiments and are relative to the control. Statistical analysis was performed using the Student's t-test. *P < 0.05, **P < 0.01, ***P < 0.001.

The similar association kinetics of cyclins D2, cyclin D3 and p27 (Figure 2F and G) suggested that p27 could play a role in the interaction of these two D-type cyclins with the promoters. Thus, we studied the binding of p27 with the D-cyclins in MEFs by IP using anti-p27 antibodies. Results revealed that in G_0_ p27 associates with all D-type cyclins and that this association clearly increased in late G_1_. IPs in p27^−/−^ MEFs were performed as a negative control (Figure 3A). ChIP analysis in p27^−/−^ MEFs versus control revealed that the association of cyclin D2 (Figure 3B) and cyclin D3 (Figure 3C) with the promoters in early and mid G_1_ was significantly reduced, indicating that p27 is responsible for the recruitment of these cyclins to the promoters after mitogenic stimulus.

### p27 is necessary for the association of Cdk4 with the promoters of target genes

**Figure 4.**
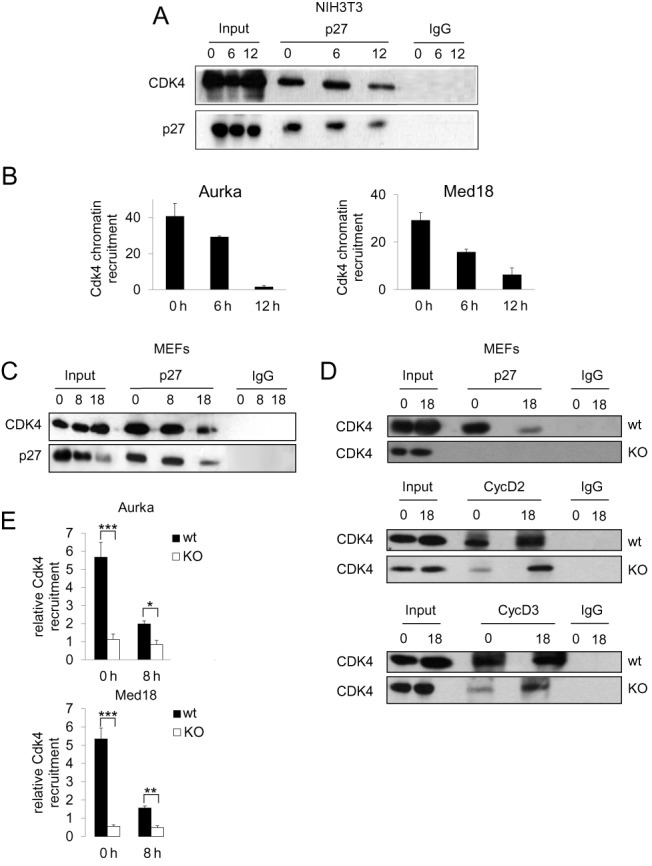
p27 is required for the recruitment of Cdk4 on the promoters of target genes. (**A**) The levels of p27 and Cdk4 at different times after proliferative activation of NIH3T3 cells were determined by WB (input). The association of p27 with Cdk4 was analyzed by IP using anti-p27. IP using a non-specific IgG was used as a control. (**B**) The association of Cdk4 to the Aurka (left panel) and the Med18 promoters (right panel) in NIH3T3 cells was analyzed by ChIP using anti-p27 or without antibodies as a control. Results are the mean value ± SD of three independent experiments and are relative to the control. (**C**) The association of p27 with Cdk4 at different times after proliferative activation of p27^WT^ MEFs was analyzed by IP using anti-p27. IP using a non-specific IgG was used as a control. (**D**) The association of p27, cyclin D2 and cyclin D3 with Cdk4 at different times after proliferative activation of p27^WT^ and p27^KO^ MEFs was analyzed by IP using anti-p27. IP using a non-specific IgG was used as a control. (**E**) The association of Cdk4 with the Aurka (upper panel) and the Med18 promoters (bottom panel) in p27^wt^ and p27^−/−^ MEFs was analyzed by ChIP using anti-p27 or without antibodies as a control. Results are the mean value ± SD of three independent experiments and are relative to the control. Statistical analysis was performed using the Student's t-test. *P < 0.05, **P < 0.01, ***P < 0.001.

As D-type cyclins are the main partners of Cdk4 we analyzed the interactions of this kinase with p27 and these cyclins. In NIH3T3, Cdk4 levels remain more or less constant during G_1_ and IP analysis revealed that the association of p27 with Cdk4 was high during G_0_ and mid G_1_ but decreased in late G_1_ (Figure 4A). ChIP studies of the association of Cdk4 to the p27-TG promoters in NIH3T3 cells revealed that its association was high in G_0_, intermediate in mid G_1_ and low in late G_1_ (Figure 4B). IP experiments in MEFs showed that the association of p27 with Cdk4 was similar to that observed in NIH3T3 (Figure 4C). We also studied the interaction of Cdk4 with p27, cyclin D2 and cyclin D3 by IP in p27^WT^ and p27^−/−^ MEFs. These experiments revealed that while the association of cyclin D2 and D3 with Cdk4 was abundant in p27^WT^ MEFs in early and late G_1_, cyclin D2 and D3 binding to Cdk4 was significantly reduced in p27-null MEFs, in agreement with p27 playing a role of assembly factor for Cdk4/D-type cyclin complexes (Figure 4D). Furthermore, ChIP analysis in p27^WT^ versus p27^−/−^ MEFs revealed that in the absence of p27, the interaction of Cdk4 with the promoters was strongly reduced (Figure 4E) indicating that p27 is necessary for the recruitment of Cdk4 to the promoters in G_0_ and mid G_1_.

### The mutant p27CK^−^ is not able to recruit cyclin D2/D3 and Cdk4 on the promoters of target genes

**Figure 5.**
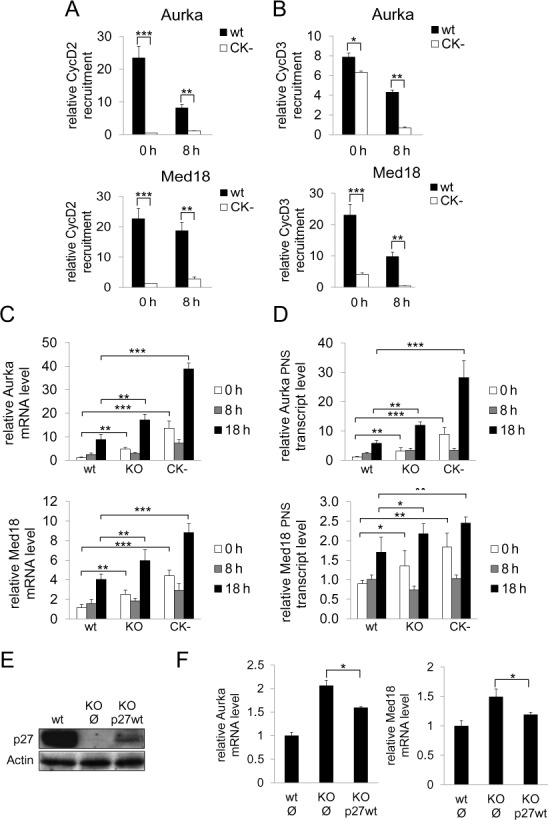
The KID domain of p27 is essential for the proper regulation of Aurka and Med18 transcription. The association of cyclin D2 (**A**) and cyclin D3 (**B**) with the Aurka (upper panels) and the Med18 promoters (bottom panels) in p27^WT^ or p27^CK−^ MEFs was analyzed by ChIP using anti-cyclin D2, anti-cyclin D3 or without antibodies as a control. Results are the mean value ± SD of three independent experiments and are relative to the control. (**C**) The levels of Aurka (upper panel) and Med18 (bottom panel) mRNAs were determined by qPCR at different times after proliferative activation in p27^WT^, p27^−/−^ and p27^CK−^ MEFs. Results are the mean value ± SE of three independent experiments and are relative to the control. (**D**) The Aurka (upper panel) and Med18 (bottom panel) primary non-spliced transcript (PNS transcript) levels were determined by qPCR in p27^WT^, p27^−/−^ and p27^CK−^ MEFs. The PNS transcript was determined using a primer of the first exon and another one of the first intron. Results are the mean value ± SD of three independent experiments and are relative to the control. (**E**) p27^−/−^ MEFs were infected with an empty vector or with a vector harboring full length p27WT. Levels of p27 in these cells were determined by WB. (**F**) The levels of Aurka and Med18 mRNAs in p27^−/−^ MEFs infected with a vector harboring full length p27WT were determined by qPCR. Results are the mean value ± SD of three independent experiments and are relative to the control. Statistical analysis was performed using the Student's t-test. *P < 0.05, **P < 0.01, ***P < 0.001.

To further confirm that p27 is responsible of the recruitment of cyclin D2, cyclin D3 and Cdk4 on the promoters of target genes we performed ChIP analysis in MEFs from animals whose p27^WT^ has been substituted by the mutant p27^CK−^. This mutant carries four point mutations in the KID domain that prevent its association with cyclins and Cdks (26). It has been previously reported that p27^CK−^ is able to associate with the p27-TG promoters because the interaction is performed by the COOH region (17). ChIP analysis performed in p27^CK−^ MEFs revealed that the association of cyclin D2 (Figure 5A) and cyclin D3 (Figure 5B) to the promoters was reduced in p27^CK-^ cells at early and mid G_1_, as observed in p27^−/−^-MEFs. These results confirm that p27 recruits cyclin D2 and D3 on the promoters via its KID domain.

### Expression of Aurka and Med18 was altered in p27^−/−^ and p27^CK-^ MEFs

Next, we studied the expression of Aurka and Med18 during G_1_ in p27^WT^, p27^−/−^ and p27^CK−^ MEFs. The mRNA levels of these two targets in p27^WT^ -MEFs were low in G_0_, intermediate in mid G_1_ and high in late G_1_ (Figure 5C). Interestingly, in p27^−/−^ and p27^CK−^ MEFs the levels of these mRNAs in G_0_ were higher than in control cells confirming that p27 is acting as a transcriptional co-repressor and that the integrity of the KID domain of p27 is necessary for the transcriptional regulation of these genes. Similarly, in late G_1_, the levels of mRNA for both genes were further increased and were much higher than in control cells (Figure 5C). To analyze whether these mRNA changes, observed in p27^−/−^ and p27^CK−^ MEFs, were due to increased transcription we determined the levels of the primary non-spliced transcript by qPCR using a primer of the first exon and another one of the first intron. Results showed that the levels of the primary non-spliced transcript were also elevated in these cells (Figure 5D) indicating that p27 represses transcription of these target genes in a Cdk-dependent manner. This was supported by experiments showing that overexpression of p27 in p27^−/−^ cells reversed the increased levels of both Aurka and Med18 mRNAs (Figure 5E).

### The association of cyclin D1 and Cdk2 with the promoters of p27 target genes is independent of p27

The observation that the association kinetics of cyclin D1 with the promoters of p27-TGs was quite different from that of cyclin D2, D3, p27 and Cdk4 (Figures 2F, G and 4B), suggested that the association of this cyclin with the promoters could be independent of p27. These data also suggested that cyclin D1 might play a role on the promoters independently of Cdk4.

**Figure 6.**
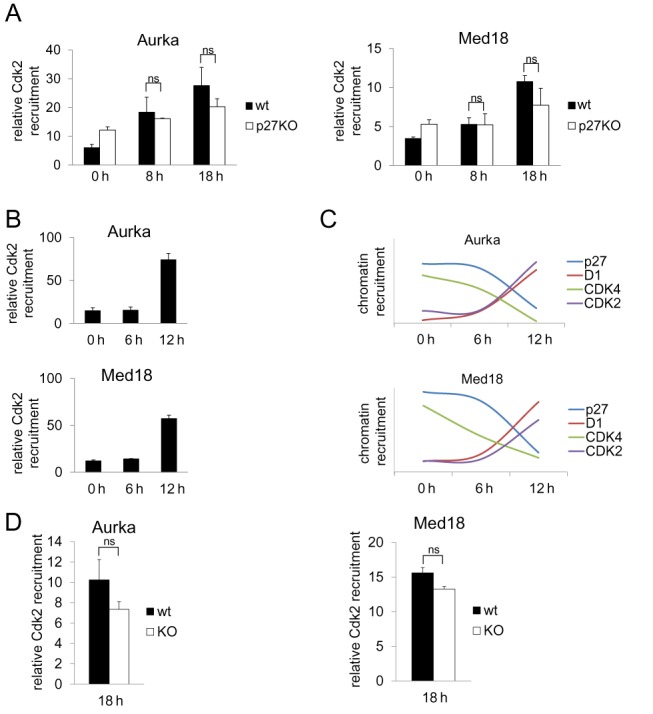
The association of Cyclin D1 and Cdk2 with the promoters of target genes in late G_1_ is independent of p27. (**A**) The association of Cdk2 with the Aurka (left panel) or the Med18 promoter (right panel) in p27^WT^ and p27^KO^ MEFs was analyzed by ChIP using anti-Cdk2 or without antibodies as a control. Results are the mean value ± SD of three independent experiments and are relative to the control. (**B**) The association of Cdk2 with the Aurka (upper panel) and the Med18 promoters (bottom panel) at different times after proliferative activation of NIH3T3 cells was analyzed by ChIP using anti-Cdk2 or without antibodies as a control. Results are the mean value ± SD of three independent experiments and are relative to the control. (**C**) Graphics showing the association kinetics of p27, cyclin D1, Cdk4 and Cdk2 with the Aurka (upper panel) and the Med18 promoters (bottom panel). (**D**) The association of Cdk2 with the Aurka (left panel) and the Med18 promoters (right panel) in late G_1_ in p27^WT^ and p27^KO^ MEFs was analyzed by ChIP using anti-Cdk2 or without antibodies as a control. Results are the mean value ± SD of three independent experiments and are relative to the control. Statistical analysis was performed using the Student's t-test. ns: not significant.

Thus, ChIP experiments were performed in p27^WT^ and p27^−/−^ MEFs. Results revealed that in the absence of p27, cyclin D1 remained associated with the promoters at early, mid and late G_1_ (Figure 6A). These results indicated that the association of cyclin D1 to the promoters was not influenced by p27, consistent with the different behavior of cyclin D1 and p27 at promoters.

We next analyzed the interaction of p27 with Cdk2 in NIH3T3 cells. We observed that it was low in G_0_, intermediate in G_1_ and high in late G_1_ (Supplementary Figure S1). Analysis of the association of Cdk2 with promoters in these cells revealed that it was low in G_0_ and in mid G_1_ but high in late G_1_ (Figure 6B). Interestingly, the kinetics of the association of cyclin D1 and Cdk2 with the promoters was very similar (Figure 6C) suggesting that cyclin D1 and Cdk2 can form complexes on the promoters. Finally, we studied whether Cdk2 association with the promoters depended of p27. For this, ChIP experiments were performed in p27^WT^ and p27^−/−^ MEFs and although the amount of Cdk2 associated with the promoters in p27^−/−^ MEFs in late G_1_ was slightly lower than in p27^WT^ MEFs (Figure 6D), this reduction was not significant. This indicates that analogously to what occurs with cyclin D1, p27 is not required to recruit Cdk2 at promoters.

### Association of cyclin D1 and Cdk2 with the promoters of target genes depends on p21

**Figure 7.**
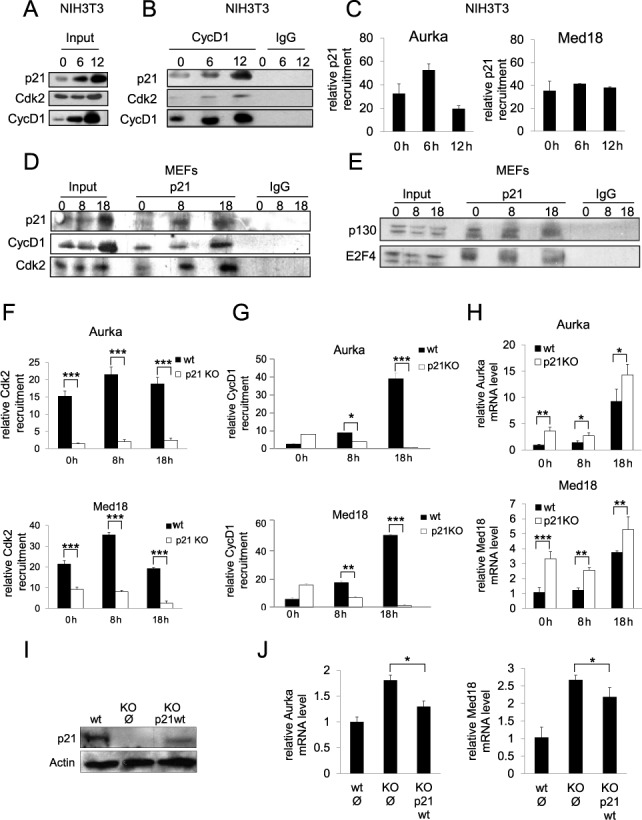
p21 is required for the association of Cyclin D1 and Cdk2 with the promoters of target genes in late G_1_. (**A**) The levels of p21, cyclin D1 and Cdk2 at different times after proliferative activation of NIH3T3 cells were determined by WB. (**B**) The interaction of cyclin D1 with p21 and Cdk2 in NIH3T3 cells was analyzed by IP using anti-cyclin D1. IP using a non-specific IgG was used as a control. (**C**) The association of p21 with the Aurka (upper panel) and the Med18 promoters (bottom panel) in NIH3T3 cells was analyzed by ChIP using anti-p21 or without antibodies as a control. Results are the mean value ± SD of three independent experiments and are relative to the control. (**D**) The levels of p21, cyclin D1 and Cdk2 along the cell cycle in p27^WT^ MEFs were determined by WB (input). The interaction of p21 with cyclin D1 and Cdk2 was analyzed by IP using anti-p21. IP using a non-specific IgG was used as a control. (**E**) The levels of E2F4 and p130 along the cell cycle in p27^WT^ MEFs were determined by WB (input). The interaction of p21 with E2F4 and p130 at different times after proliferative activation was analyzed by IP using anti-p21. IP using a non-specific IgG was used as a control. (**F**)The association of Cdk2 with the Aurka (upper panel) and the Med18 promoters (bottom panel) in late G_1_ in control and p21^KO^ MEFs was analyzed by ChIP using anti-Cdk2 or without antibodies as a control. Results are the mean value ± SD of three independent experiments and are relative to the control. (**G**) The association of cyclin D1 with the Aurka (upper panel) and the Med18 promoters (bottom panel) in late G_1_ in control and p21^KO^ MEFs was analyzed by ChIP using anti-cyclin D1 or without antibodies as a control. Results are the mean value ± SD of three independent experiments and are relative to the control. (**H**) The levels of Aurka (upper panel) and Med18 mRNAs (bottom panel) were determined by qPCR at different times after proliferative activation in control and p21^KO^ MEFs. Results are the mean value ± SD of three independent experiments and are relative to the control. (**I**) p21^−/−^ MEFs were infected with an empty vector or with a vector harboring full length p21WT. Levels of p21 in these cells were determined by WB. (F) The levels of Aurka and Med18 mRNAs in p21^−/−^ MEFs infected with a vector harboring full length p21WT were determined by qPCR. Results are the mean value ± SD of three independent experiments and are relative to the control. Statistical analysis was performed using the Student's t-test. *P < 0.05, **P < 0.01, ***P < 0.001.

We explored the possibility that Cdk2 and cyclin D1 could form a complex at promoters in late G_1_. We observed that in NIH3T3 cells the levels of cyclin D1 were low in G_0_, increased in mid G_1_ being highest in late G_1_ with an accumulation kinetics similar to that of p21 (Figure 7A). In contrast, the levels of Cdk2 remained constant along cell cycle. IP experiments in these cells showed that the interaction of cyclin D1 with Cdk2 and p21 was low in G_0_, intermediate in mid G_1_ and high in late G_1_ (Figure 7B). ChIP analysis with anti-p21 antibodies revealed that in these cells, the association of p21 with the Med18 promoter was relatively constant along the cell cycle but showed slight fluctuations in the association with the Aurka promoter (Figure 7C). In wild-type MEFs, the expression of cyclin D1, Cdk2 and p21 and their interaction was similar to that observed in NIH3T3 cells (Figure 7D). In p21^−/−^-MEFs levels of Cdk2 remained constant along cell cycle, whereas those of cyclin D1 were increasing similarly to control. However, in p21^−/−^-MEFs the amount of cyclin D1 was significantly lower than controls (Supplementary Figure S2) as also observed in p27^−/−^-MEFs (Figure 3A). IP experiments using anti-p21 antibodies revealed the interaction of this protein with E2F4 and p130 (Figure 7E) indicating that similarly to p27, p21 could associate to promoters via E2F4/p130 complexes. The possibility that p21 could be responsible of cyclin D1 and Cdk2 recruitment on the promoters was analyzed by ChIP performed in control and p21^−/−^ MEFs. ChIP analyses revealed that the association of Cdk2 (Figure 7F) and cyclin D1 (Figure 7G) with the promoters depended on the presence of p21. To confirm the importance of p21 in the regulation of the expression of the target genes, we determined the levels of their mRNAs during G_1_ progression in control and p21^−/−^ MEFs. We found that in all cases the levels of both mRNAs in p21^−/−^ -MEFs was higher than in control MEFs (Figure 7H). These results revealed that p21 behaves as a transcriptional repressor of these target genes. This was further supported by experiments showing that overexpression of p21 in p21^KO^ cells reversed the increased levels of both Aurka and Med18 mRNAs (Figure 7I and J).

### p27 and p21 collaborate in the repression of target genes

**Figure 8.**
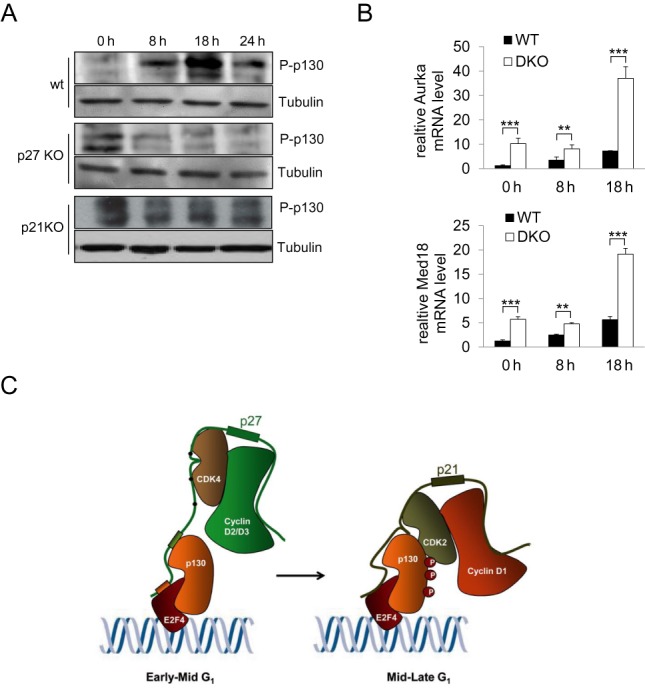
p27 and p21 collaborate in the repression of target gene expression. (**A**) The levels of phosphorylated p130 at different times after proliferative activation in control, p27^−/−^ and p21^−/−^ MEFs were determined by WB using anti-phospho-p130. The amount of tubulin was detected with anti-tubulin and was used as a loading control. (**B**) The levels of Aurka (upper panel) or Med18 mRNAs (bottom panel) were determined by qPCR at different times after proliferative activation in control and the double p27/p21 null MEFs (DKO). Results are the mean value ± SD of three independent experiments and are relative to the control. (**C**) Model of the sequential role of p27 and p21 in the regulation of transcription. Statistical analysis was performed using the Student's t-test. **P < 0.01, ***P < 0.001.

As p27 and p21 sequentially recruit different cyclin–cdk complexes on the promoters of target genes we studied whether the absence of these proteins would facilitate the phosphorylation of p130, a well-known substrate of these cyclin–cdk complexes that is present in the repressor complexes operating on the promoters during the G_1_ phase of the cell cycle. We analyzed the phosphorylation status of p130 during G_1_ in control, p27 and p21 null MEFs, by using anti-phospho-p130 antibodies. As shown in Figure 8A in control MEFs p130 started to be phosphorylated at 8 h (mid G_1_) reaching a maximum at 18 h (late G_1_). Interestingly, in both p27 or p21 null cells, p130 phosphorylation was already high at time 0 that corresponds to G_0_. Then, the levels of phosphorylation decreased and remained lower (Figure 8A). These results revealed that p27 and p21 participate in the regulation of transcription by controlling p130 phosphorylation.

The fact that p27 and p21 associate with the same promoter regions and that they act as transcriptional repressors of the same genes suggested that they can collaborate in the regulation of gene expression. To confirm this possibility we studied the expression of Aurka and Med18 in MEFs lacking both p27 and p21 (DKO). As shown in Figure 8B, these cells showed levels of mRNA of these two genes higher than control cells but also higher than the single knock out cells for p27 or p21 (Figures 5C and 7G). These results indicate that p27 and p21 collaborate in a sequential manner in the repression of transcription during G_1_.

## DISCUSSION

Previous work described that p27 regulates transcription by associating with p130/E2F4 complexes on the promoters of specific p27-TGs (17). p27 is a structural component of these repressor complexes that operate in quiescent cells and in the G_1_ phase of the cell cycle to repress the expression of genes encoding for proteins needed for DNA replication or mitosis (17). These p27-TGs include genes involved in cell cycle progression, respiration, translation and RNA processing and splicing (17). A recent report also revealed a role of p27 in reprogramming mouse fibroblasts into pluripotent stem cells by regulating the expression of Sox2 through p130/E2F4 complexes (21). However, the specific role of p27 in this regulation remained unknown.

p27 directly interacts with E2F4 and p130 via its carboxy-terminal region (17). In contrast, its interaction with cyclins and Cdks is mediated by the KID domain located at the N-terminus of the protein (27). Thus, p27 could simultaneously associate with both cyclin–Cdks and p130/E2F4 repressor complexes on the promoters. As p130 is directly phosphorylated by different Cdks (28), the interaction of p27 with both types of complexes on the transcriptional regulatory regions of the genes would allow it to timely regulate cyclin–Cdk activity and as a consequence p130 phosphorylation and transcription. We explored here this possibility.

We found that p27 associated with cyclins D2, D3 and Cdk4 on the promoters of specific p27-TGs in quiescent fibroblasts (NIH3T3 and MEFs). This association was maintained till mid G_1_. The recruitment of cyclin D2/D3–Cdk4 was dependent on p27 because it was impaired in p27 null cells. Since the mutant p27^CK-^ also associated with the promoters (17) the lack of binding of cyclins D2/D3 and Cdk4 at these specific sites in cells containing p27^CK−^ confirmed that p27 recruits cyclin D2/D3–Cdk4 at the promoters through its KID domain.

Phosphorylation of p130 in mid G_1_ suggested that the previously inactive cyclin D2/D3–Cdk4 complexes were activated at that time. The mechanism responsible for this activation on the promoters still remains unclear. However, an attractive possibility is that they can be activated by phosphorylation of p27 on Y74 and Y88 by members of the Src family of tyrosine kinases. Indeed, phosphorylation of p27 at these sites induces a conformational change that leads to the partial activation of the Cdk despite the presence of p27 associated to cyclin–Cdk complexes (29). This possibility is currently under study in our laboratory. Phosphorylation of p130 by cyclin D2/D3–Cdk4 complexes primes it for its subsequent phosphorylation by Cdk2.

Interestingly, we found that in late G_1_, p27, cyclin D2, cyclin D3 and Cdk4 were no longer present at the promoters, seemingly to be replaced by cyclin D1–Cdk2 complexes. The recruitment of Cdk2 and cyclin D1 was independent of p27 but required p21 since p21 null cells were unable to accumulate these proteins on the promoters in late G_1_. The interaction of p21 with E2F4 and p130 observed by IP indicates that the association of p21 to promoters could be via E2F4/p130, similarly to p27. Thus, the sequential association of cyclin D2/D3–Cdk4 in early–mid G_1_ and of cyclin D1–Cdk2 complexes in late G_1_ at gene promoters is driven by p27 and p21, respectively. By sequentially recruiting these cyclin–Cdk complexes p27 and p21 play role in transcriptional regulation of these genes (Figure 8C).

We observed that in the absence of p27, p130 phosphorylation was advanced and it was already phosphorylated in early G_1_ (Figure 8A). This phosphorylation was concomitant with a significant increase of transcription of target genes also seen at that time (Figure 5C and D). These results indicate that p27 defines the timing of p130 phosphorylation. In control cells p27 on the promoters keeps Cdk4 inhibited till mid G_1_ when the shift from inactive to active Cdk4 was produced. In contrast, in p27 null cells cyclin D2/D3–Cdk4 complexes were already active in early G_1_ and despite they were not associated to the promoters they phosphorylated p130 at that time by transiently interacting with the substrate. We also report here a similar role for p21. The fact that in the double p21/p27 null cells the expression of target genes was much higher than in single knock-out or control cells confirmed the collaborative role of p27 and p21 in the regulation of transcription.

All these results clarify the role of p27 and p21 in the transcriptional regulation of genes, repressed by p130/E2F4, mainly involved in the regulation of DNA replication and mitosis during cell cycle progression. We still do not know whether this is a general mechanism operating in all the p130/E2F4 regulated genes but the evidence that p27 and p21 bind to E2F4/p130 suggest that it could be the case. This collaboration also suggests that at least some transcriptional programs regulated by p27 might be the same as those regulated by p21.

Overall, in cells undergoing a reversible cell cycle arrest, the transcriptional repression machinery (p130/E2F4) recruits CKIs at promoters which in turn can sequentially recruit, while inhibiting them, cyclin–CDK complexes. The assembly of these repressor/activator complexes on promoters of genes needed for cell cycle progression offers a versatile mechanism for the cell to rapidly resume a proliferative state.

Increasing evidence indicate that p27 and p21 can act both as tumor suppressors or oncogenes during tumor development (29–33). Thus, results reported here revealing the mechanisms by which both proteins collaborate in the regulation of transcription of genes involved in cell cycle progression might open new insights into the participation of both proteins in tumorigenesis.

## SUPPLEMENTARY DATA

Supplementary Data are available at NAR Online.

SUPPLEMENTARY DATA
