# Supplementary material for: p27Kip1 and p21Cip1 collaborate in the regulation of transcription by recruiting cyclin–Cdk complexes on the promoters of target genes
Source: Nucleic Acids Res. 2015 Jun 13;43(14):6860–73. doi: 10.1093/nar/gkv593 (PMC4538812; doi:10.1093/nar/gkv593)
Supplement: SUPPLEMENTARY DATA [file supp_43_14_6860__index.html]

p27Kip1 and p21Cip1 collaborate in the regulation of transcription by recruiting cyclin–Cdk complexes on the promoters of target genes — SUPPLEMENTARY DATA 

# p27Kip1 and p21Cip1 collaborate in the regulation of transcription by recruiting cyclin–Cdk complexes on the promoters of target genes

## SUPPLEMENTARY DATA

- SUPPLEMENTARY DATA
