# Supplementary material for: p27Kip1 and p21Cip1 collaborate in the regulation of transcription by recruiting cyclin–Cdk complexes on the promoters of target genes
Source: Nucleic Acids Res. 2015 Jun 13;43(14):6860–73. doi: 10.1093/nar/gkv593 (PMC4538812; doi:10.1093/nar/gkv593)
Supplement: SUPPLEMENTARY DATA [file supp_gkv593_nar-03691-x-2014-File010.pdf]

Supplementary Table S1

qRT-PCR primers	sequence (5'-3')
Aurka FWD	TCAAACCCTCTATCCAAGAGC
Aurka REV	CTTACTCACTGGCACGTTCG
Med18 FWD	ACAGGAAACTTCTCCGCTACCG
Med18 REV	TAGGACCCCAAAGCAACAGCAG
Aurka 1st transcript FWD	CCCATTCCCACAAGAACCTA
Aurka 1st transcript REV	AAACGGATAGGGAAGGCTGT
Med18 1st transcript FWD	GGGACACGTGTGCACTTACA
Med18 1st transcript REV	GTGCTGAAGGGAGAGTTTGG

ChIP primers	sequence (5'-3')
Aurka ChIP FWD	AGTTCCTGATGGTGGTCCAG
Aurka ChIP REV	TTTCTGGCCTGCTGGATAGT
Med18 ChIP FWD	TAGGAAGGTGAGGGACAGGA
Med18 ChIP REV	CTAGGACCCCAAAGCAACA

Primers used for qPCR and ChIP

Supplementary Figure S1

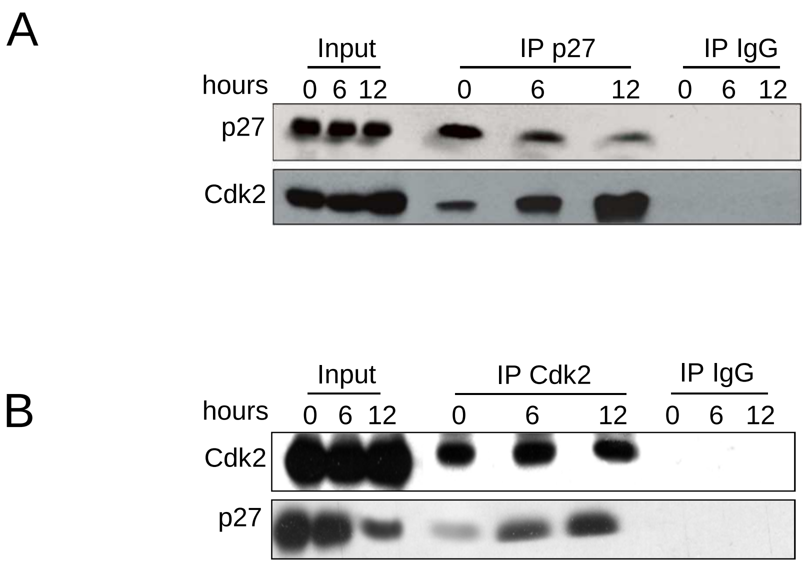

Supplementary Figure S1. Association of p27 and Cdk2 during G_1 in NIH3T3 cells . (**A**) The levels of p27 and Cdk2 at different times after proliferative activation of NIH3T3 cells were determined by WB (input). The association of p27 with Cdk2 during cell cycle was analyzed by IP using anti-p27. IP with a non-specific IgG was used as a control. (**B**) The levels of p27 and Cdk2 at different times after proliferative activation of NIH3T3 cells were determined by WB (input). The association of Cdk2 with p27 during cell cycle was analyzed by IP using anti-Cdk2. IP with a non-specific IgG was used as a control.

Supplementary Figure S2

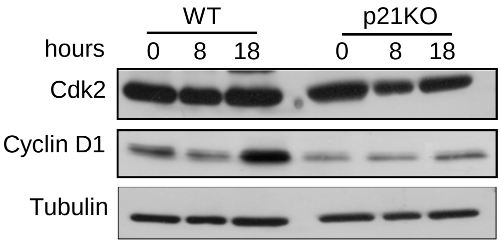

Supplementary Figure S2. Levels of cyclin D1 and Cdk2 during G_1. The levels of Cdk2 and cyclin D1 at different times after proliferative activation in WT or p21 knock out (KO) MEFs were determined by WB. WB with anti-tubulin was used as a loading control.
